# Oral extracellular vesicles in early pregnancy can identify patients at risk of developing gestational diabetes mellitus

**DOI:** 10.1371/journal.pone.0218616

**Published:** 2019-06-26

**Authors:** Lara J. Monteiro, Manuel Varas-Godoy, Max Monckeberg, Ornella Realini, Marcela Hernández, Gregory Rice, Roberto Romero, José F. Saavedra, Sebastián E. Illanes, Alejandra Chaparro

**Affiliations:** 1 Department of Obstetrics and Gynecology, Centre for Biomedical Research, Faculty of Medicine, Universidad de los Andes, Santiago, Chile; 2 Department of Periodontology, Centre for Biomedical Research, Faculty of Dentistry, Universidad de los Andes, Santiago, Chile; 3 Department of Pathology, Faculty of Dentistry, Universidad de Chile, Santiago, Chile; 4 Dentistry Unit, Faculty of Health Sciences, Universidad Autónoma de Chile, Santiago, Chile; 5 Centre for Clinical Research, University of Queensland, Herston, Qld, Australia; 6 Perinatology Research Branch, Program for Perinatal Research and Obstetrics, Division of Intramural Research, Eunice Kennedy Shriver National Institute of Child Health and Human Development, NIH, Bethesda, Maryland and Detroit, United States of America; 7 Department of Obstetrics and Gynecology, University of Michigan, Ann Arbor, MI. Department of Epidemiology and Biostatistics, Michigan State University, East Lansing, MI. Center for Molecular Medicine and Genetics, Wayne State University, Detroit, Michigan, United States of America; 8 Health Family Centre CESFAM Karol Wojtyla, Santiago, Chile; Ospedale dei Bambini Vittore Buzzi, ITALY

## Abstract

**Aim:**

To isolate and characterize oral extracellular vesicles from gingival crevicular fluid at 11–14 weeks and evaluate their capacity to identify patients at risk of developing gestational diabetes mellitus.

**Methods:**

A case-control study was conducted, including patients who developed gestational diabetes mellitus (n = 11) and healthy pregnant controls (n = 23). Obstetric and periodontal histories were recorded at 11–14 weeks of gestation, and samples of gingival crevicular fluid obtained. Extracellular vesicles were isolated from gingival crevicular fluid by ExoQuick. Nanoparticle tracking analysis, ELISA and transmission electron microscopy were used to characterize extracellular vesicles.

**Results:**

Total extracellular vesicles isolated from gingival crevicular fluid were significantly higher in patients who developed gestational diabetes mellitus later in pregnancy compared to normoglycemic pregnant women (6.3x10^9^ vs 1.7 x10^10^, *p* value = 0.0026), and the concentration of the extracellular vesicles delivered an area under the ROC curve of 0.81. The distribution size of extracellular vesicles obtained using ExoQuick was around 148 ± 57 nm. There were no significant differences in the periodontal status between cases and controls. The exosome transmembrane protein CD63 was also detected in the extracellular vesicles of gingival crevicular fluid.

**Conclusion:**

We were able to isolate extracellular vesicles from gingival crevicular fluid using a method that is suitable to be applied in a clinical setting. Our results provide an insight into the potential capacity of first trimester oral extracellular vesicles as early biomarkers for the prediction of gestational diabetes mellitus in pre-symptomatic women.

## Introduction

Gestational Diabetes Mellitus (GDM) is defined by glucose intolerance of various degrees with primary identification during pregnancy [1, 2]. The global occurrence of hyperglycemia in pregnancy has risen to 17 percent in recent years, fluctuating between 10% in North America and 25% in Southeast Asia [3, 4]. The main contributing factors to the global burden of this disease are aging of the population, suburbanization, rates of overweight and obesity among pregnant women, sedentary habits and stress of contemporary life [1, 3, 5]. Pregnancies complicated with GDM are expected to develop type 2 diabetes mellitus over the next 10 to 30 years [5, 6]. Moreover, their offspring are at higher risk of developing short-term adverse complications such as macrosomia, neonatal hypoglycemia and neonatal cardiac dysfunction, but also long-term problems such as obesity, impaired glucose tolerance, and diabetes in puberty or in early adulthood [3, 7].

The criteria for the diagnosis of GDM were initially established more than 40 years ago and, with minor modifications, remains in use until today. Current management guidelines recommend “universal screening” for GDM at 24–28 weeks of gestation by oral glucose tolerance tests [7–9]. In patients with positive screening, two randomized trials show beneficial results for both the mother and the offspring, with treatment [10]. The management of this disorder either with dietary intervention, self-monitoring of blood glucose or with insulin therapy, significantly reduced the risks of fetal overgrowth, shoulder dystocia, cesarean delivery, and hypertensive disorders [7, 11, 12]. Although a glucose challenge test at 24–28 weeks is diagnostically robust, it has some disadvantages. Firstly, it is time consuming for clinician and patient and presents false positive rate [13–15]. The second disadvantage of the 24–28 weeks oral glucose challenge test is that it does not facilitate early treatment of GDM. Hence the fetus is exposed to an unmodified adverse hyperglycemic environment for the whole of the first and part of the second trimester. Current efforts to reduce the burden of the disorder have been focused on early identification of patients at risk of developing GDM to allow interventions to reduce the prevalence of the disease and its long-term impact in both, mother and fetus [7].

In the past few years, periodontal chronic infection, a common disease among pregnant women, has emerged as a risk factor for GDM [16]. In fact, the prevalence of chronic periodontitis is higher in women with GDM (44.8%) in comparison with non-diabetic pregnant women (13.2%), with an adjusted odds ratio (aOR) of 9.11 (95% confidence interval: 1.11–74.9) [17, 18]. Even though the biological mechanism involved behind the association between periodontitis and GDM remain to be elucidated, the release of inflammatory mediators [including, tumor necrosis factor alpha (TNF-α), interleukin-6 (IL-6), and C-reactive protein (CRP)] from inflamed periodontal tissues that are known to interfere with glucose metabolism by inducing insulin resistance, has biological plausibility [19, 20]. Therefore, periodontal pockets could represent, during pregnancy, a permanent source of IL-6, CRP and TNF-α that may affect the insulin signaling and consequently increase glucose intolerance, and increase the risk of GDM.

Recently, extracellular vesicles (EVs) have been suggested as a liquid biopsy for the diagnosis and prognosis of different kind of pathologies, since they are released from a variety of tissues, including the placenta, into the circulation [21]. Particularly, exosomes, a group of small EVs are released from the placenta and can be detected in plasma as early as 6 weeks of gestation and their concentration during the first trimester is increased in patients that develop GDM later in pregnancy [22, 23]. Interestingly, recent studies have shown that these EVs are present in several body fluids, including oral fluids as saliva [24]. Gingival crevicular fluid (GCF), another type of oral fluid, is a serum exudate and/or transudate originated in the gingival sulcus that is exacerbated by the inflammation of periodontal tissues. It transports molecular biomarkers filtered from the systemic and local circulation, and as we have published before [25–27], the GCF can be used as a suitable source for biomarkers since it concentrates systemic circulation content and can be collected in a convenient and minimally-invasive manner. A proteomic analysis of GCF has demonstrated that most of the proteins in this oral fluid derive from extracellular exosomes [28], however, until now, there is no characterization of EVs in GCF.

The aims of the present study were to determine whether the concentration and/or size distribution of EVs present in GCF are altered during the first trimester of pregnancies that later develop GDM; and to assess if the periodontal inflammation status is related to the concentration and/or size distribution of EVs in GCF.

## Materials and methods

### Study design

A case control study was conducted in the family health center, CESFAM Karol Wojtyla, Santiago, Chile. Enrolment and clinical, physical and obstetric data were collected at 11–14 weeks of gestation. Pregnant women were evaluated for gestational diabetes at 24–28 weeks of gestation (oral glucose tolerance test) and retrospectively stratified into two groups: GDM and healthy controls. From the prenatal cohort (n = 215) all of the GDM cases (n = 11) were selected at the moment of GDM diagnosis. The control group (n = 23) was randomly selected using the same cohort, and matched for age, socioeconomic status and body mass index.

A complete dental evaluation and full-mouth periodontal examinations were performed by a qualified periodontist, with a high intra-examiner reliability (0.88–0.91 kappa test) at 11–14 weeks. This study was reviewed and approved by the Universidad de los Andes Scientific Ethics Committee before the study began. A written informed consent was also approved by this institutional review board. All patients participating in the study have been properly instructed and have indicated that they consent to participate by signing the appropriate informed consent form. The sample size was arbitrarily established according to the number of patients enrolled with GDM in the cohort. The variables studied were GDM, glycemia, blood pressure, periodontal clinical measures and diagnoses and number of teeth.

### Diagnoses criteria

GDM was diagnosed at 24–28 weeks of gestation using the oral glucose tolerance test (OGTT): fasting plasma glucose levels ≥ 92 mg/dL, and/or plasma glucose levels 2h after oral administration of 75g glucose ≥ 153 mg/dL, according to the IADPSG criteria [8]. Both the control and the GDM groups were singleton pregnant women. Moreover, the control group did not present any chronic medical conditions or obstetric complications.

Women with periodontitis were identified using the following criteria: at least four teeth with 4 mm or higher probing pocket depth (PPD), with 3 mm or higher clinical attachment loss (CAL), and inflammation and bleeding on probing (BOP). Women with more than 20% of the sites with BOP and gingival redness, and without CAL, were diagnosed as gingivitis. Women with PPD < to 4 mm, CAL < 3mm, and less than 25% of the sites with BOP were classified as healthy.

### GCF collection and elution

GCF samples were collected at 11–14 weeks of gestation. Briefly, the supragingival plaque was removed using curettes without contacting the marginal gingiva, and the gingival sulcus was then dried gently with an air syringe. GCF was collected using Periopaper strips (Oraflow, Smithtown, NY, USA) placed into the sulci/pocket for 30 s and isolated from 4 periodontal pockets (1 x quadrant) of the most representative periodontal site. Representative samples were then stored in 1.5 mL tubes at -80°C until elution. Strips contaminated by saliva and blood, were discarded. For elution of GCF, 4 Periopaper strips (Oraflow, Smithtown, NY, USA) were placed in a 1.5 mL tube containing 160 μL of phosphate buffer saline (PBS) (Corning, Mediatech Inc, NY, USA) and protease inhibitor cocktail (EDTA Complete, mini, EDTA-free Protease Inhibitor Cocktail, Roche, USA). Tubes were vortexed and incubated on ice for 30 min, and then centrifuged at 12,000 x g for 5 min at 4°C. The eluate was collected and placed on ice. The elution procedure was repeated and both eluates were pooled and stored at -80°C until analysis.

### EVs isolation from GCF

EVs from GCF were isolated by precipitation with the commercial reagent ExoQuick (System Biosciences Inc., Mountain View, CA, USA) according to the manufacturer’s recommendations. In brief, 300 μL of GCF eluate was mixed with 150 μL of ExoQuick reagent and incubated overnight at 4°C. The day after, the ExoQuick-GCF complex was centrifuged at 1,500 x g for 30 min at room temperature to obtain the EVs precipitate that was subsequently suspended in 200 μL of PBS.

### Nanoparticle tracking analysis

The size distribution and concentration of EVs were analyzed by Nanotracking particle analysis (NTA) using NanoSight NS300 instrument (Malvern, Worcestershire, UK). Non-diluted EVs isolated from GCF were evaluated and their size, distribution and concentration were determined.

### Transmission electron microscopy

The EVs isolated from GCF were assessed by transmission electron microscopy (TEM) in the TEM facility of the Faculty of Biological Sciences (Pontificia Universidad Católica de Chile, Santiago, Chile), Briefly, 5 μL of EVs suspension were diluted 10 times in PBS and deposited on Formvar-carbon coated electron microscopy grids and left to adsorb for 20 min. The grids were then stained with 5% uranyl acetate for 5 min and washed with distilled water. After drying for 5 min at 60°C the grids were examined in the Phillips CM100 TEM at 80 kV.

### Quantification of CD63 by ELISA

EVs were isolated from GCF by ExoQuick as mentioned above and total protein concentration was measured using Qubit Protein Assay kit (ThermoFisher Scientific). The presence of the exosome membrane marker, CD63, was identified by ELISA, using the EXOEL-CD63A-1 kit (System Biosciences, Palo Alto, CA, USA). The protocol applied was according to the manufacturer’s instructions.

### Statistical analyses

For numerical variables, gaussian distribution was tested using Shapiro-Wilk normality test, and homogeneity of variances was tested using variance ratio test. For variables, fitting gaussian distribution, comparisons among groups was performed using Students T-test with correction for unequal variances where necessary. For variables that did not fit normal distribution, Mann Whitney U-Tests were performed for comparisons. To assess the diagnostic performance of extracellular vesicles concentration, receiver operating characteristic (ROC) curves analyses were performed. Empirical estimation of optimal cut-point was obtained using the Youden index method [29]. Sensitivity, specificity and positive and negative predictive values were calculated. A two tailed *p*-value < 0.05 was considered statistically significant. The statistical package used was STATA v.14.2. (StataCorp. 2015. Stata Statistical Software: Release 14. College Station, TX: StataCorp LP). Graphing was performed using GraphPad Prism 6.0.

## Results

### Demographic and clinical characteristics of the study population

The clinical characteristics of the population at the time of enrollment are summarized in Table 1. As expected from the matched criteria, patients who developed GDM during their pregnancies and the normoglycemic group presented similar mean maternal ages (27.1 years old for controls *vs* 28.3 years old) and similar body mass index (BMI) (28.3 Kg/m^2^ for controls *vs* 28.5 Kg/m^2^ for GDM). As seen in Table 1, there are no significant differences between systolic and diastolic blood pressure and the weight of patients from both groups. Nevertheless, the control group presented statistically higher height compared to GDM group (*p*-value = 0.037). Regarding the periodontal characteristics of the study population, the median number of teeth present was the same in both groups (n = 28) (Table 2). Moreover, no significant differences were observed in the different periodontal clinical parameters evaluated in both groups, such as: plaque index (*p* value = 0.898), bleeding on probing (BOP) (*p* value = 0.994), periodontal pockets probing depth mean (PPD) (*p* value = 0.750) and clinical attachment level (CAL) mean (*p* value = 0.472) (Table 2).

**Table 1:** Description of demographic and clinical characteristics of healthy pregnant women (Control group) and Gestational Diabetic Mellitus pregnant women (GDM group) at 11–14 weeks of gestation.

Variables	Control group (n = 23)	GDM group (n = 11)	
	Median (p25—p75)	Median (p25—p75)	P-value
Height (m)	1.58 (1.56–1.61)	1.54 (1.52–1.57)	0.037^1^
Weight (kg)	70.3 (58.5–80.0)	70.0 (51.5–82.2)	0.664^1^
OGTT (fasting glucose, mg/dL)	85 (81–88)	97 (93–109)	< 0.0001^1^
OGTT (2h glucose, mg/dL)	105 (101–118)	160 (154–175)	< 0.0001^1^
Blood Pressure (mmHg):			
Systolic	110 (98–110)	110 (100–118)	0.618^1^
Diastolic	60 (60–70)	64 (54–70)	0.863^1^
Nutritional State, n (%):			0.185^2^
Normal	7 (30.4)	4 (36.4)	
Overweight	8 (34.8)	2 (18.2)	
Obese	8 (34.8)	5 (45.5)	

Results are expressed in median (P50) with interquartilic range (P25-P75).

^1^ = Mann–Whitney Test

^2^ = Fisher Exact Test

GDM, Gestational Diabetes Mellitus; m, meters; kg, kilograms; OGTT, 75-g oral glucose tolerance test (milligrams/decilitre); mmHg, millimetre of mercury.

**Table 2:** Description of periodontal characteristics of control group (healthy pregnancies) and Gestational Diabetic Mellitus pregnant women (GDM group) at 11–14 weeks of gestation.

Variables	Control group (n = 23)	GDM group (n = 11)	
	Median (p25—p75)	Median (p25—p75)	P-value
Number of teeth	28 (27–28)	28 (27–28)	0.868
Periodontal probing depth (mean, mm)	2.8 (2.5–3.3)	2.8 (2.3–3.0)	0.750
Clinical Attachment Level (mean, mm)	2.3 (1.9–2.9)	2.2 (1.9–2.5)	0.472
Plaque Index (% of sites)	83 (64–98)	60 (46–82)	0.092
Bleeding on probing (% of sites)	79 (49–96)	54 (21–78)	0.099

Results are expressed in median (P50) with (P25-P75). Statistical significance, p < 0.05, Unpaired Student T-Test, Mann-Whitney Test. GDM, Gestational Diabetes Mellitus; mm, millimetre.

### Characterization and quantification of EVs in GCF

**Fig 1:**
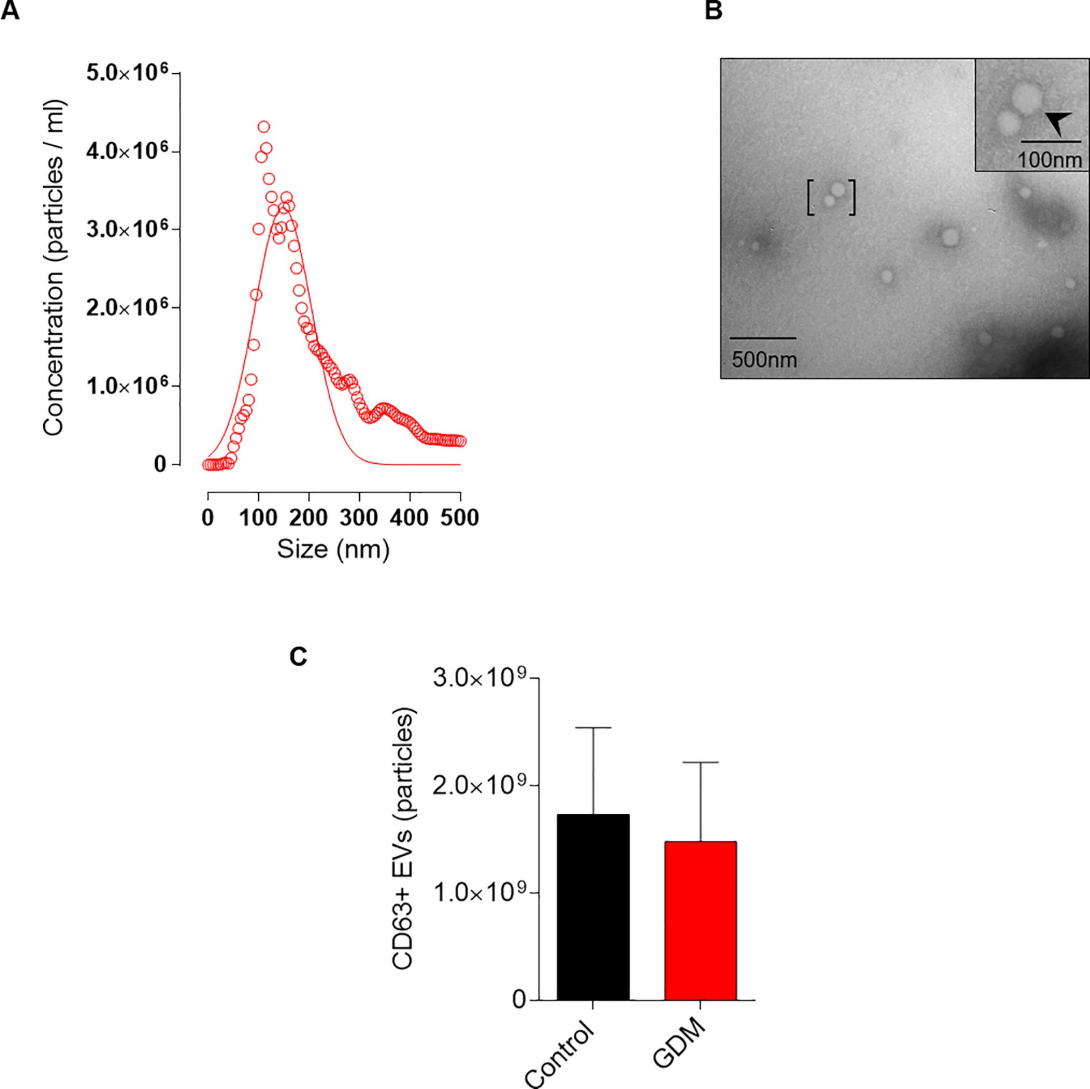
Characterization of EVs isolated from GCF using ExoQuick in controls and patients with GDM. **A.** Mean particle diameter of EVs obtained from GCF for all the samples by nanoparticle tracking analysis. **B.** Representative image of transmission electron microscopy of EVs obtained. **C.** CD63 positive EVs obtained from total EVs isolated from GCF were analyzed by ELISA.

We next sought to characterize the EVs isolated from GCF using the ExoQuick. Nanoparticle tracking analysis clearly demonstrates that the size distribution of GCF-derived EVs was enriched at 148 ± 57 nm (Fig 1A). Morphological analysis identified the presence spherical vesicles (Fig 1B) and CD63 ELISA analysis (intra-assay variability <6.22%) of this fraction confirmed the presence of the transmembrane marker, CD63, characteristic of EVs [30] (Fig 1C). Altogether, these results suggest that the GCF-derived EVs resemble microvesicles [30].

**Fig 2:**
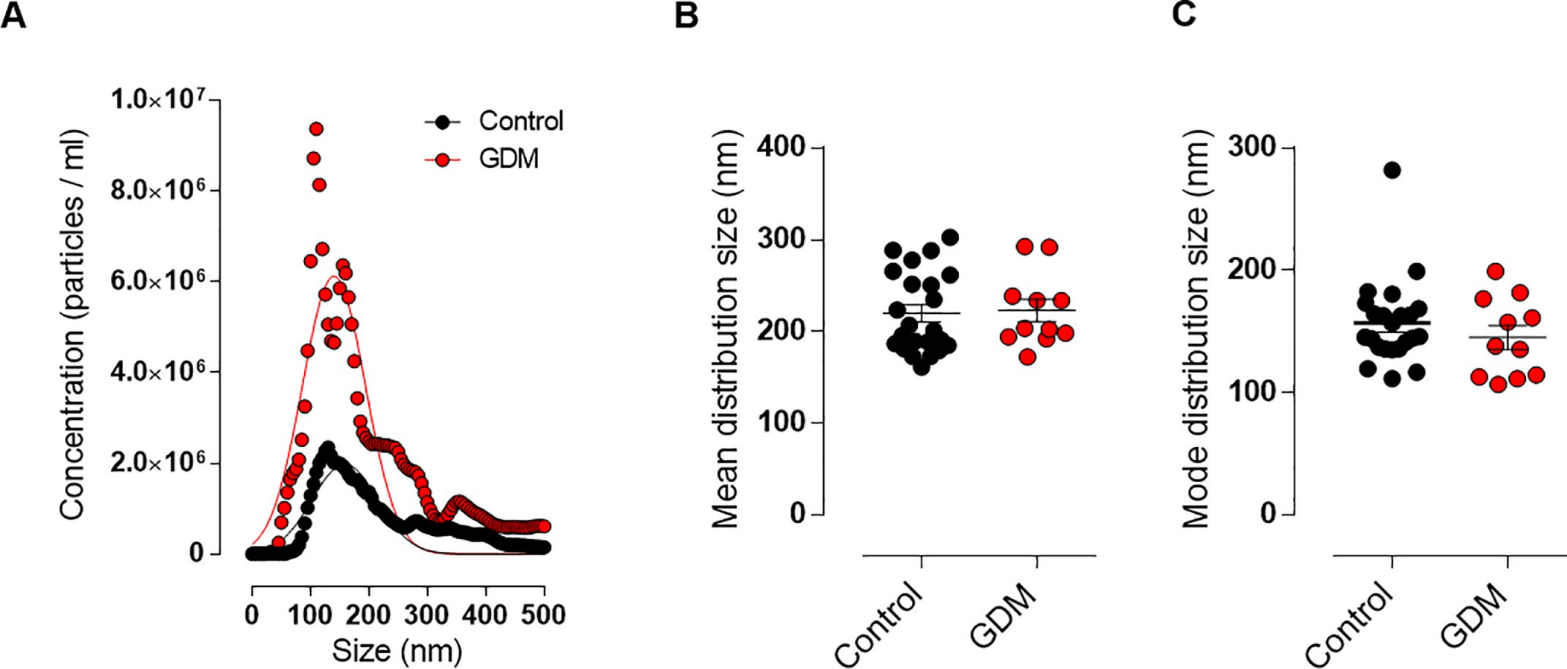
Nanoparticle tracking analysis of EVs isolated from GFC using ExoQuick in controls and patients with GDM. **A.** Distribution size of EVs isolated from GCF with higher concentration of nanoparticles in the GDM group (red dots) in comparison with the control group (black dots). **B.** Mean and **C.** Mode distribution size of total extracellular vesicles isolated from Controls (n = 23) and GDM (n = 11) pregnant women. GDM, gestational diabetes mellitus.

**Fig 3:**
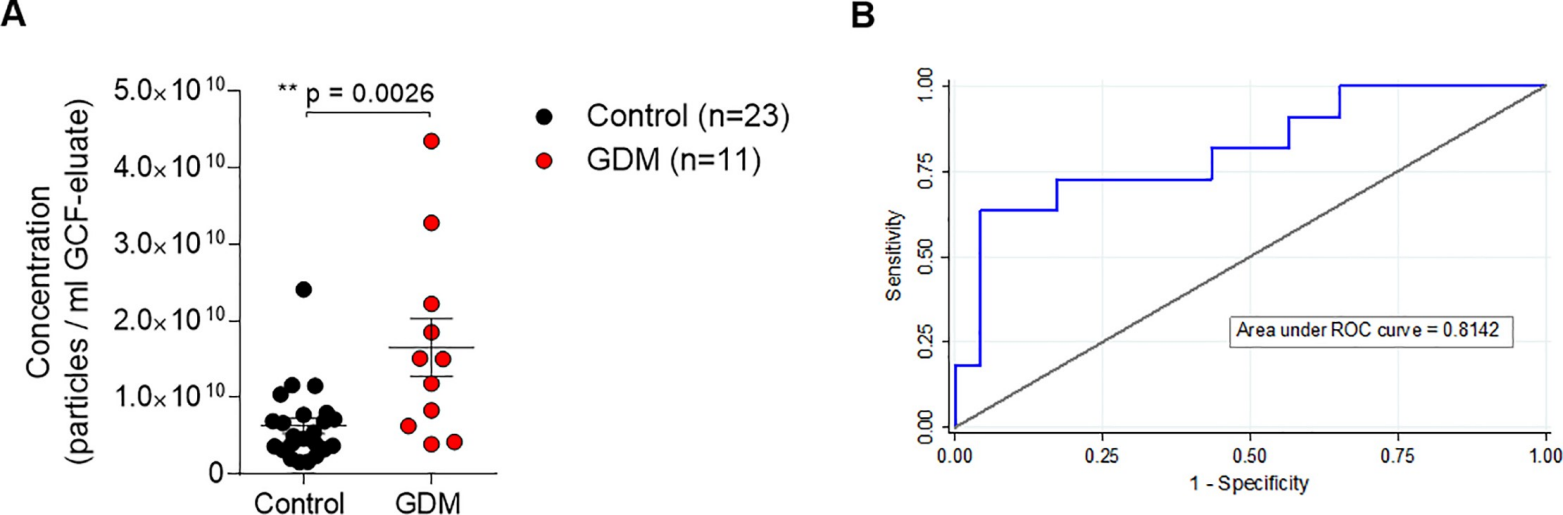
Concentration of EVs isolated from GCF in Controls and GDM patients. **A.** Mean concentration of total extracellular vesicles present in GCF eluate from Controls (n = 23) and GDM (n = 11). Results are the mean±SEM. ***p ≤ 0.001. GDM, gestational diabetes mellitus. **B.** Receiver Operating Characteristic curve for the prediction of GDM by log_10_ EVs concentration in the first trimester (AUC = 0.814). AUC, area under the curve.

To analyze whether there were differences in GCF-derived EVs between the GDM and control groups in early pregnancy, we further analyzed the EVs-size distribution of both groups. There was no significant difference in the size distribution of the EVs between GDM and Controls, as demonstrated by the similar Gaussian curves and the mean and mode distribution size graphs (Fig 2A, 2B and 2C). Nevertheless, the mean concentration of microvesicles was significantly higher in GCF obtained from pre-symptomatic GDM women when compared with patients with euglycemic pregnancies (controls 6.3x10^9^ vs GDM 1.7 x10^10^, *p* value = 0.0026) (Fig 3A). To evaluate the diagnostic accuracy of using EVs concentration as a biomarker for early prediction of GDM, ROC curve analysis was performed. The observed area under the ROC curve was 0.8142 (95% CI: 0.650–979) (Fig 3B). The empirical optimal cut point level of extracellular vesicles was calculated at a threshold of 1.17 x 10^10^ particles/ml in GCF eluate. At this cut point, EVs demonstrated a sensitivity of 63.6%, specificity of 95.7%, and positive and negative predictive values of 87.5% and 84.6%, respectively, for the prediction of GDM.

## Discussion

Exosomes are a subpopulation of extracellular vesicles that are ~40–120 nm in size and, since they have endosomal origin, exosomes are enriched with late endosomal membrane markers, including TSG101, CD63, CD9, and CD81 [30]. The EVs obtained from GCF, however, were slightly bigger and, are therefore, considered microvesicles, which are defined as EVs of ~100–1000 nm produced by fragmentation of plasma membrane, released into extracellular environment and that are characterized by CD40 enrichment [30]. Thus, to our knowledge, this study identifies for the first time the presence of CD63 positive microvesicles with a mean diameter of 148 ± 57 nm in GCF. Moreover, the data obtained support the potential prognostic utility of a simple GCF sample collection and microvesicles concentration analysis to identify women at risk of developing GDM during the first trimester of pregnancy.

Ultracentrifugation is the “gold standard” method to obtain minimally contaminated EVs [31], however it demands a very prolonged process utilizing specialized equipment that is not usually found in most clinical laboratories. Moreover, since the volume of GCF eluate is limited, it is not possible to isolate EVs from this fluid using ultracentrifugation. Thus, in order to establish a prognostic performance and clinical utility of EVs in oral fluids, it is important to select more suitable methods to be used in the clinical setting. The methodology of the precipitation of EVs used in ExoQuick is regarded as a good alternative to ultracentrifugation, especially when dealing with limited volume of samples, which is the case of GCF. Furthermore, using ExoQuick-isolated EVs it was sufficient to allow us to distinguish between patients that are going to develop GDM later in their pregnancies and those who will remain euglycemic through pregnancy.

Nanoparticles present in GCF may be released from junction and sulcus of epithelial cells or inflammatory cells, may be derived from circulating nanoparticles, or may represent a combination of both sources. The identification of nanoparticle-associated immunoreactive CD63 protein and the presence of ~150 nm diameter nanovesicles may be consistent with the presence of exosomes in GCF samples.

The importance of the study of EVs in a clinical setting to complement the diagnosis and prognosis of several diseases has been well demonstrated [32–34]. Nonetheless, it is important to highlight that the election of the most appropriate technique to be used in the clinic depends on the required outcome, which could be: to obtained the highest concentration of EVs, to select one particular type of EVs (i.e. exosomes, microvesicles, or apoptotic bodies), or to get the less time-consuming, less labor-intensive and more economic protocol [35]. Furthermore, EVs could have a series of advantages when compared to other biomarkers, including, stability on storage, and the possibility to separate them from high abundance proteins from plasma that have been a problem for prediction models using proteomics in the past [21, 36]. If identification of specific profiles of EVs in GCF in first trimester of pregnancy in patients that will develop GDM can be achieved, this may provide an opportunity for early stratification of risk and the implementation of clinical interventions that may prevent the occurrence of the pathology for improvement of the outcomes for the mother and offspring. The goal of developing antenatal screening tests to predict and prevent pathologies in pregnancy as GDM, is not only to improve the management of the pregnancy but also to optimize lifelong and intergenerational health.

Several reports have demonstrated an association between periodontitis and increased risk of GDM [16–18]. Although the exact mechanism involved in the association between periodontitis and GDM remains unclear, the fact that periodontitis can contribute to the systemic spread of bacteria and bacterial products, and subsequently induce a systemic inflammatory process, that is related with the physiopathology of GDM, makes this association biologically plausible. However, in this study, we did not find significant differences between the periodontal clinical parameters in controls and GDM. On the other hand, oral fluids can be used as a surrogate source of plasmatic biomarkers as we have demonstrated recently for Placental Growth Factor (PlGF) [27].

In the last few years, we have explored the role of biomarkers in the periodontal tissue, specifically in GCF [25–27]. GCF is an inflammatory exudate secreted at the gingival margin, the periodontal pocket, or both, and it is composed of serum as well as different cell types such as leukocytes, desquamated epithelial cells, periodontium cells, bacteria and their byproducts, enzymes derived from the subgingival biofilm, and inflammatory mediators secreted by the host [27, 37–39]. To our knowledge this is the first time that EVs have been characterized in GCF, opening up new opportunities for their use as biomarkers for the prediction of GDM.

In a normal pregnancy, insulin resistance increases during the late second trimester [1]. However, most women will remain euglycemic because of higher insulin secretion due adequate beta cell compensation [3]. GDM will develop if this beta-cell compensation is insufficient for the insulin resistance and if there is a lack of hepatic glucose production driven by placental diabetogenic hormones [7]. EVs and/or exosomes could act as key information vectors between elevated glucose and the development of GDM [36, 40]. Indeed, we have recently shown that the number of placenta-derived exosomes in maternal plasma is higher in overweight and obese women (BMI >30 kg/m^2^) [22, 23]. Circulating nanoparticles may have pathogenic effects on vascular thrombosis, vascular inflammation, and angiogenesis and also promotes the interaction between endothelia cells and monocytes [41, 42]. For example, in the placenta, exosome-derived trophoblastic cells are able to reprogram monocytes to secrete specific cytokine profiles independent of cell-to-cell contact [43]. Indeed, trophoblastic derived exosomes induce pro-inflammatory cytokine such IL-1B in human macrophage cells [44–46]. Probably, higher amounts of EVs and exosomes in GCF reflect a possible mechanism that link GDM with periodontal inflammation.

## Conclusion

Here we report, for the first time, the isolation and characterization of EVs from GCF using a method that can be easily applied in the clinical setting. Moreover, the higher concentration of EVs obtained at 11–14 weeks of gestation from women that went on to develop GDM during their pregnancies, compared to normoglycemic controls, suggests that in pregnancies that will be complicated by GDM, a hyperglycemic and pro-inflammatory state stimulates the release of EVs in oral fluids early in pregnancy. The quantification of EVs in GCF of pre-symptomatic women, alone or in combination with other factors or clinical history of the patients, could potentially be used as a first trimester screening. Indeed, if an effective early screening test was available, the damage accumulated during the clinically occult phase (i.e. up to 24–28 weeks) could provide an opportunity for the establishment or prevention and/or treatment programs, improving the outcomes for both mother and offspring. In sum, these are the main strengths of this study. Nevertheless, we consider that the main limitation is that the study was conducted on a small number of patients.

In addition to this preliminary pilot study, we need to explore the EVs content, to determine their potential biological effects in the GDM development and periodontal tissues inflammatory status.
